# Analgesic efficacy of blocking nerve to vastus lateralis muscle versus lateral femoral cutaneous nerve after knee surgeries: a randomized trial

**DOI:** 10.1186/s12871-026-03680-8

**Published:** 2026-03-07

**Authors:** Sherif Kamal Arafa, Wafaa Madhy Abdelwahed, Ahmed Shama, Ahmed Mhamed Elattar, Mahmoud Mohammed Mustafa

**Affiliations:** 1 Anesthesiology, Surgical Intensive Care and Pain Medicine Department, Faculty of Medicine, Kafr Elsheikh University, Kafr Elsheikh, 33511 Egypt; 2 Anesthesiology, Surgical Intensive Care and Pain Medicine Department, Faculty of Medicine, Tanta University, Tanta, Egypt; 3 Orthopedic Consultant at Al-Ahrar Teaching Hospital, General Organization for Teaching Hospitals and Institutes (GOTHI), Cairo, Egypt; 4 Anesthesia, Faculty of Medicine, Cairo University, Cairo, Egypt

**Keywords:** Vastus lateralis, Lateral femoral cutaneous nerve, Knee surgery, Satisfaction, Analgesia

## Abstract

**Background:**

Postoperative pain is common following knee surgeries. Effective pain management is crucial for speeding up recovery, improving satisfaction for patients, and reducing the risk of complications. This research aimed to assess analgesic effectiveness and functional outcomes of blocking the nerve to the vastus lateralis (NVL) muscle versus the lateral femoral cutaneous nerve (LFCN).

**Methods:**

This randomized, double-blind study was carried out on 80 patients aged ≥ 18 years, both sexes, who underwent knee surgeries under spinal anesthesia using 15 mg of bupivacaine 0.5%. Patients were allocated to two equal groups: Group VL was given the NVL block, and Group LFCN received the LFCN block. Blocks were performed at the surgical ending under ultrasound guidance with 5 ml of 0.5% bupivacaine. The primary outcome was the numerical rating score.

**Results:**

Time of onset of sensory block and time required to achieve the maximum sensory block, pain scores at 4, 6, and 12 h postoperatively, and total morphine consumption were significantly lower, while time to the first request for rescue analgesia was significantly postponed in Group NVL compared to Group LFCN (*P* < 0.001). Patient satisfaction, side effects, and hospital stay lengths were similar between both groups, with no cases of respiratory depression or local anesthetic toxicity.

**Conclusions:**

The NVL block may serve as a potential alternative to the LFCN block for postoperative analgesia in knee surgeries, as both techniques provided comparable postoperative pain control and were safe and well tolerated, while the faster sensory onset and reduced opioid consumption associated with the NVL block did not translate into a meaningful improvement in pain control.

**Clinical trials registration:**

Registered at clinicaltrials.gov (ID: NCT06809842, Date of registration: 30/01/2025, URL: https://clinicaltrials.gov/study/NCT06809842?cond=NCT06809842&rank=1).

## Background

Knee surgeries are correlated with substantial postoperative pain, that may impact early mobilization and prolong recovery [1].

Postoperative pain following knee surgery is multifactorial, arising from both nociceptive and inflammatory mechanisms. The surgical procedure itself leads to tissue trauma, inflammation, and activation of pain pathways, contributing to significant discomfort in the immediate and subacute postoperative periods [2].

Poorly controlled pain can hinder physical therapy participation, delay hospital discharge, and ultimately compromise functional recovery [3]. Effective pain management strategies are crucial to enhance patient outcomes and reduce the incidence of chronic pain following knee surgeries [1].

Traditionally, various regional anesthesia techniques, such as femoral nerve blocks, have been utilized to mitigate post-knee surgery pain [4]. However, the optimized analgesic efficacy is often coupled with quadriceps muscle weakness, causing minimal motor impairment that led to the exploration of alternative nerve blocks [5].

To mitigate motor impairment, alternative nerve blocks targeting specific sensory innervations have been explored [6]. Recent advances in ultrasound-guided regional anesthetic have facilitated more targeted nerve blocks, such as the blockade of the nerve to vastus lateralis (NVL) [7] and the lateral femoral cutaneous nerve (LFCN) [8].

The VL nerve specifically innervates the vastus lateralis muscle (VLM), which is a significant contributor to knee joint stability and postoperative pain. Blocking NVL may offer targeted analgesia while preserving quadriceps strength [9].

Conversely, the LFCN provides sensory blockade to the anterolateral thigh without affecting motor function, making it a potential option for postoperative analgesia in knee surgeries [10].

Despite these theoretical advantages, there is limited clinical evidence comparing the analgesic efficacy of LFCN blocks versus blocks of the nerve to the VLM following knee surgeries. Existing studies have primarily focused on the femoral nerve and adductor canal blocks, with few directly evaluating these more selective nerve blocks [11].

We hypothesized that NVL would provide superior analgesic efficacy compared to LFCN in patients undergoing knee surgeries. By targeting distal branches to the vastus lateralis and adjacent peri-articular tissues, the NVL block may better address the deep, movement-related pain that limits early mobilization while largely preserving quadriceps strength. Therefore, we expect NVL to produce better analgesia, and improved early functional recovery and patient satisfaction compared with LFCN.

Thus, this study evaluated the analgesic efficacy and functional outcomes of blocking the NVL versus the LFCN in cases experiencing knee surgeries.

## Methods

This randomized, double-blind study involved 80 cases, aged ≥ 18 years, both sexes, American Society of Anesthesiologists (ASA) physical status from I to III who underwent knee surgeries under spinal anesthesia and were admitted to Tanta University Hospitals. The trial was conducted from February 2025 to July 2025 following approval from Ethical Committee, Faculty of Medicine, Kafr Elsheikh University, Kafr Elsheikh, Egypt (approval code: MKSU 50-1-4) and registered at clinicaltrials.gov (ID: NCT06809842, Date of registration: 30/01/2025, URL: https://clinicaltrials.gov/study/NCT06809842?cond=NCT06809842&rank=1). The study procedures follow the guidelines in the World Medical Association (WMA) Declaration of Helsinki. A written informed consent was obtained from all subjects participating in the trial, before study commencement.

Pregnancy, coagulopathy, neuromuscular disorders, hematological disorders, mental disorders, history of multiple traumas or anesthesia drug allergies, body mass index (BMI) greater than 40, local skin infection at the block site, and opioid analgesics or abusing opioids were excluded.

### Randomization and blindness

To maintain the integrity of the study, a random allocation process was utilized, employing computer-generated numbers using an online website (https://www.randomizer.org/). Each participant’s code was placed in an opaque, sealed envelope to preserve blinding. Individuals were distributed at random into two groups (1:1 ratio) to underwent either NVL block in Group VL or LFCN block in Group LFCN. Both cases and outcome assessors were blinded to the allocation.

Prior to the surgical procedure, all participants underwent a comprehensive review of their medical history, clinical examination, and laboratory testing. Furthermore, they were familiarized with the numerical rating scale (NRS) for pain assessment to ensure that they could accurately report their pain level.

Both groups received 0.03 mg/kg intravenous midazolam after an 18-G intravenous (IV) cannula was inserted. This study’s standard patient monitoring included temperature probe, noninvasive blood pressure, ECG, and pulse oximetry.

Spinal anesthesia was administered using 15 mg of bupivacaine 0.5% via a 25G needle at the L2–L3 or L3–L4 intervertebral space.

The blocks were carried out by a single anesthesiologist under sterile conditions at the end of surgery. The ultrasound (SonoScape ^®^ (A6, Shenzen, China) transducer used for the block was a high-frequency linear type (5–10 MHz).

### Technique of blocking the nerve to the vastus lateralis muscle

The patient was positioned supine with the knee extended. The femoral artery was identified and traced distally. The LCFA was typically situated between the rectus femoris and VL muscles, in a linear pattern from the anterior superior iliac spine (ASIS) to the lateral edge of the patella, aligning with the lateral intermuscular septum. The distal branch of the NVL, positioned laterally to the LCFA, was clearly visible. The needle was introduced at the lateral side of the thigh then advanced under ultrasound guidance toward the NVL, where up to 5 ml of 0.5% bupivacaine was injected after confirming correct needle placement with a negative aspiration test.

### Technique of lateral femoral cutaneous nerve block

The transducer was positioned parallel to the inguinal ligament just below the ASIS. The Sartorius muscle (SaM) and tensor fascia lata muscle (TFLM) were identified. In a short-axis view, the lateral femoral cutaneous nerve appeared as a small hypoechoic oval structure with a hyperechoic rim, located superficial to the SaM. The needle was inserted between the TFLM and SaM, and after confirming proper needle positioning through negative aspiration, 5 ml of 0.5% bupivacaine was injected.

A pinprick test with a 25-G hypodermic needle was employed to assess the sensory block level at the midclavicular line at one-minute intervals until the peak block was attained and subsequently at 15-minute intervals until the block regressed to the S3 dermatome.

### Postoperative

Patients were transferred to the PACU. A standardized analgesic regimen was prescribed for the postoperative period. All patients were given 1 g of paracetamol every 6 h as routine analgesia. If the NRS exceeded 3, rescue analgesia administered as a 3 mg morphine bolus was administered, to be repeated after 30 min if the pain persisted until the NRS dropped below 4. NRS assessments were conducted at the PACU, and at 2, 4, 6, 12, 18, and 24 h postoperatively.

Side effects were documented, including postoperative nausea and vomiting (PONV), respiratory depression, and local anesthetic (LA) toxicity. Management of potential side effects, such as hypotension (regarded as a decrease in systolic blood pressure ≥ 30% from baseline or SBP < 80 mmHg) and bradycardia (heart rate ≤ 50 beats/min), was as follows: hypotension was managed with 5 mg of ephedrine, and bradycardia was addressed with 0.5 mg of intravenous atropine.

Patient satisfaction 24 h post-intervention was measured using a 5-point Likert scale (1 = extremely dissatisfied, 2 = unsatisfied, 3 = neutral, 4 = satisfied, 5 = extremely satisfied). Also, the length of hospital stay (up to one week) was recorded from admission to discharge.

The primary outcome was the NRS, while secondary outcomes included time to first request for analgesia, total morphine consumption within 24 h, block performance time, patient satisfaction, hospital length of stay, and adverse events. The minimum clinically important difference (MCID) for the NRS was predefined as a 1.5-point reduction in pain score, based on previous literature, to distinguish clinically meaningful improvement from statistical variation [12].

### Sample size calculation

The sample size was determined utilizing G. Power 3.1.9.2 (Universitat Kiel, Germany), aiming for an α error of 0.05 and 80% power. The calculation was based on a 30% reduction in NRS at 4 h for Group VL compared to Group LFCN (3.9 ± 2.0) [13]. Four additional cases were added to each group to account for potential dropouts, resulting in 40 patients per group.

### Statistical analysis

Utilizing SPSS v27 (IBM, Armonk, New York, United States), the data were examined. The Shapiro-Wilk test as well as histogram were utilized in order to evaluate whether or not the data were normal. Non-parametric data are reported as median and interquartile range (IQR), and they were evaluated using the Mann-Whitney test. Parametric data are provided according to the meaning plus the standard deviation (SD), and the unpaired t-test was used to investigate them. Both the Chi-square test and Fisher’s exact test were used in order to conduct the analysis on the categorical variables, which are provided as frequencies (percent). To determine statistical significance, a p-value of less than 0.05 was considered significant.

## Results

**Fig. 1:**
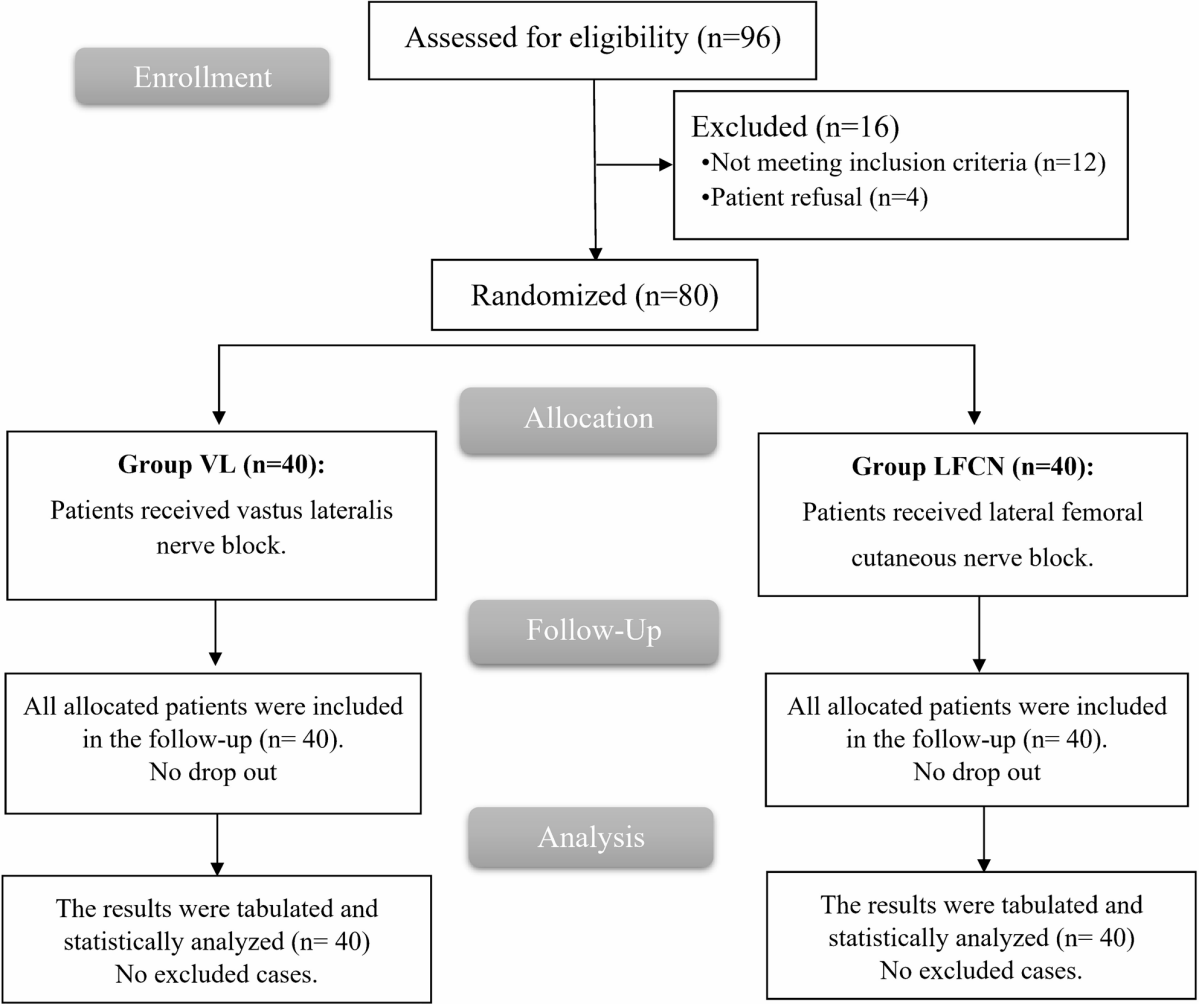
CONSORT flow chart

This investigation initially assessed 97 patients for eligibility, with 80 ultimately participating after 12 failed to meet the criteria of inclusion and five rejected to engage in the trial. These patients were randomly allocated into two groups and continued follow-up and analysis (Fig. 1).

Demographic characteristics, surgery duration, block performance times and time of surgery exhibited no significant differences between the groups (Table 1).

**Table 1:** Demographic characteristics, duration of surgery, block performance time and type of surgery of the studied groups

P	Group LFCN(n=40)	Group VL(n=40)		
0.365	49.13 ± 14.78	46.1 ± 14.93	Age (years)	
0.648	17 (42.5%)	15 (37.5%)	Male	Sex
	23 (57.5%)	25 (62.5%)	Female	
0.216	82.35 ± 14.26	85.95 ± 11.42	Weight (kg)	
0.658	166.93 ± 5.54	167.5 ± 6.03	Height (cm)	
0.293	29.59 ± 5.1	30.72 ± 4.37	BMI (kg/m^2^)	
0.236	39.75 ± 7.42	41.88 ± 8.45	Duration of surgery (min)	
0.170	5.2 ± 3.95	4.18 ± 2.52	Block performance time (min)	
0.966	11 (27.5%)	10 (25%)	Meniscus Repair	Types of surgery
	13 (32.5%)	15 (37.5%)	Arthroscopic meniscectomy	
	9 (22.5%)	9 (22.5%)	Chondroplasty	
	7 (17.5%)	6 (15%)	Synovectomy	

Data are presented as mean ± SD or frequency (%). BMI: Body mass index

Time of onset of sensory block and time required to achieve the maximum sensory block were significantly lower in Group VL than Group LFCN (*P* < 0.001). Sensory block duration and motor block duration were significantly higher in group VL than group LFCN (*P* < 0.001). At 2, 18, and 24 h, NRS scores were similar between the groups, but Group VL demonstrated significantly lower NRS scores at 4, 6, and 12 h compared to Group LFCN (*P* < 0.05). Group VL also had a significantly delayed time to first rescue analgesia and a lower total morphine consumption in the first 24 h compared to Group LFCN (*P* < 0.001) (Table 2).

**Table 2:** Analgesia of the studied groups

P	Group VL(n=40)	Group LFCN(n=40)		
<0.001	286.33 ± 32.05	390.15 ± 40.56	Duration of sensory block (min)	
<0.001	298.48 ± 30.84	401.75 ± 40.53	Duration of motor block (min)	
---	0 (0 - 0)	0 (0 - 0)	0 h	NRS
0.187	1 (0 - 1)	1 (0 - 1)	2 h	
0.001	3 (2 - 3)	2 (1 - 3)	4 h	
0.033	3 (2 - 5)	2 (2 - 4)	6 h	
0.006	4 (2.75 - 5)	2 (1 - 4)	12 h	
0.097	3.5 (2.75 - 5)	3 (2 - 4)	18 h	
0.290	4 (2 - 5)	3 (2 - 4)	24 h	
<0.001	6.23 ± 1.39	7.8 ± 1.51	Time to first request of rescue analgesia (h)	
<0.001	8.03 ± 1.72	6.15 ± 1.66	Total dose of morphine consumption in the first 24 hours (mg)	

Data are presented as median (IQR) or mean ± SD. NRS: Numeric rating scale

No significant differences were demonstrated in bradycardia, hypotension, PONV, patient satisfaction, or hospital stay between the groups. Neither respiratory depression nor LA toxicity occurred in either group (Table 3).

**Table 3:** Side effects, patient satisfaction and hospital stay of the studied groups

P	Group LFCN(n=40)	Group VL(n=40)		
0.615	1 (2.5%)	3 (7.5%)	Bradycardia	Side effects
0.431	2 (5%)	5 (12.5%)	Hypotension	
0.517	7 (17.5%)	4 (10%)	PONV	
---	0 (0%)	0 (0%)	Respiratory depression	
---	0 (0%)	0 (0%)	LA toxicity	
0.324	18 (45%)	24 (60%)	Extremely satisfied	Patient satisfaction
	11 (27.5%)	11 (27.5%)	Satisfied	
	7 (17.5%)	4 (10%)	Neutral	
	4 (10%)	1 (2.5%)	Unsatisfied	
	0 (0%)	0 (0%)	Extremely dissatisfied	
0.285	3 (2-4)	2.5 (2-4)	Hospital stays (days)	

Data is presented as frequency (%) or median (IQR). PONV Postoperative nausea and vomiting, LA Local anesthetic

## Discussion

The NVL block and the LFCN block operate through different mechanisms, which may explain the variation in their analgesic efficacy. The NVL block targets the nerve to the VL, a branch of the femoral nerve that innervates the VLM, part of the quadriceps group, and may interrupt both motor and sensory fibers, which could account for altered pain perception in the anterolateral thigh and knee. This nerve block likely interrupts both motor and sensory fibers, which may have contributed to the reduced perception of pain in the anterolateral aspect of the thigh and knee. The blockade of motor fibers might also play a role in decreasing pain by limiting muscle contractions that could otherwise exacerbate postoperative pain. Blocking NVL may disrupt pain pathways involved in transmitting nociceptive signals from the knee joint and could produce more extensive or longer-lasting analgesia than purely cutaneous blocks in some patients [14].

The LFCN block, on the other hand, targets the LFCN, which is a sensory nerve originating from the lumbar plexus (L2-L3). It supplies the skin over the lateral thigh without affecting deeper structures or motor function. The primary mechanism involves blocking sensory input from the skin, thereby reducing superficial pain sensations [15]. However, since the LFCN does not innervate the deeper tissues or the knee joint itself, the analgesic effect is more limited, especially for pain originating from deeper structures involved in knee surgeries [16].

Before passing through the inguinal ligament, the LFCN typically originates from L2 and L3, with some variation in its upper distribution. It typically descends between the SaM and the TFLM origin.

In over 95% of individuals, the LFCN extends from the ASIS to the midpoint of the outer margin of the patella. The LFCN terminal branches are uniformly distributed over the upper and lateral compartments of the knee joint. Additionally, nearly 100% of individuals have an anterior branch that supplies the patella [17, 18].

The novelty of this study lies in its direct comparison of the analgesic efficacy and functional outcomes of the NVL block versus the LFCN block in knee surgeries. Also, this investigation is pioneering in its use of the NVL block for knee surgery, a technique that has not been previously explored in the literature. By introducing the NVL block as a novel approach to regional anesthesia for knee surgeries, this research provides valuable insights into its potential advantages over the more commonly used LFCN block, offering a new avenue for optimizing postoperative pain management.

We observed that the time of onset of sensory block, the time required to achieve extremely sensory block, pain scores at 4, 6, and 12 h postoperatively, and total morphine utilization were significantly lower in Group VL than in Group LFCN, while the time to the first request for rescue analgesia was significantly extending. Patient satisfaction, side effects, and hospital stay times were comparable across the two groups, with no incidences of respiratory depression or local anesthetic toxicity.

The NVL block demonstrated faster sensory block onset and quicker accomplishment of maximal sensory block compared to the LFCN block. This is likely due to the NVL’s anatomical role in innervating both sensory and motor fibers in the VLM, contributing to its ability to interrupt pain transmission from deeper muscular and periarticular structures involved in knee movement and postoperative pain [19].

The results obtained of the study can be attributed to the anatomical and functional differences between the two nerves. The NVL innervates VLM, which is a part of the quadriceps group responsible for knee extension. Blocking the NVL likely interrupts pain transmission from deeper muscular and periarticular structures that are heavily involved in knee movement and postoperative pain [20]. This comprehensive blockade of both muscular and joint-related pain pathways may result in more effective pain relief, which explains the lower pain scores and reduced morphine consumption observed in Group VL [21].

In contrast, the LFCN primarily responsible for sensory innervation to the skin of the lateral thigh, meaning that its blockade predominantly reduces superficial pain without significantly affecting deeper musculoskeletal pain [22]. The delayed time to the first request for rescue analgesia in the NVL block also suggests that the NVL blockade provides a more prolonged and potent analgesic.

Similarly, Li and co-authors [23] demonstrated that combining LFCN with adductor canal block (ACB) offers superior pain control, reduced opioid consumption, and an extended analgesia duration compared to peri-articular infiltration. This combination also preserves muscle function without influencing the hospital stay length, patient satisfaction, adverse events, or functional rehabilitation. The inclusion of LFCN may further enhance the analgesic effect of ACB by broadening the block coverage following total knee arthroplasty (TKA), thus supporting its clinical applicability.

Also, Singleton et al. [14]. reported that a multimodal analgesic regimen incorporating modified IPACK, VLN, vastus intermedius, and anterior femoral cutaneous nerve block effectively decreased the duration of moderate pain while not prolonging the period of severe pain. This approach facilitated early ambulation and led to lower opioid consumption.

Although statistically significant differences in postoperative pain scores were observed between the NVL and LFCN groups at 4, 6, and 12 h, the magnitude of these differences warrants careful interpretation regarding the MCID. The mean between-group differences in NRS pain scores at these time points were modest and consistently below the MCID threshold of 1.5 points proposed by Kindrick et al. [24] which represents the smallest change in pain intensity perceived by patients as clinically meaningful. Consequently, despite statistical significance, the observed reductions in pain scores in the NVL group may not have translated into a perceptible improvement in pain experience from the patient’s perspective.

This interpretation is further supported by findings from Laigaard and colleagues [25] who reported that an MCID of approximately 15 mm on a 100-mm visual analog scale (VAS) is commonly assumed in orthopedic surgery trials. When the observed NRS differences in the present study are extrapolated to the VAS scale, they remain well below this clinically meaningful threshold. These findings suggest that the analgesic advantage of the NVL block over the LFCN block, while statistically demonstrable, is unlikely to represent a clinically relevant superiority based on pain intensity outcomes alone.

The distinction between statistical and clinical significance is particularly important in regional anesthesia research, where large sample sizes and low variability can detect small numerical differences that may not influence patient-centered outcomes.

Similar observations have been reported in studies comparing more extensive nerve blocks with selective or sensory-only techniques. Cuñat et al. [26] demonstrated that despite broader neural coverage with femoral nerve block compared to adductor canal block, pain scores and opioid consumption did not differ in a clinically meaningful manner. Likewise, Gadsden and colleagues [27] reported non-inferior pain outcomes between genicular nerve block and periarticular infiltration despite measurable numerical differences.

This study had several limitations, including being performed at a single center, which may restrict the generalizability of the results to broader populations or settings. The relatively short follow-up period (24 h) restricted the ability to assess long-term outcomes of the analgesic techniques. Additionally, the study excluded certain patient groups, such as those with severe obesity (BMI > 40), opioid use or abuse, and various medical conditions, which may have influenced the results if included. The sample size, while calculated to notice a variance in pain scores, might have been underpowered to detect differences in less common adverse events. The clinical significance of the observed disparities in pain scores should be interpreted judiciously. The nerve block may be most appropriately viewed as a potentially beneficial element within a multimodal analgesic approach, rather than as a definitively superior, singular technique for the management of postoperative pain following knee arthroplasty.

We recommend considering the NVL block as a preferred regional anesthesia technique for knee surgeries, particularly when optimizing postoperative pain management and minimizing opioid use. Future research should explore the efficacy of the NVL block in various surgical procedures beyond knee surgeries, such as hip surgeries and anterior cruciate ligament (ACL) reconstruction, to determine its broader applicability. Additionally, comparing the NVL block with other established nerve blocks, such as the adductor canal block or femoral nerve block, would help clarify its relative benefits and limitations. Further investigations should also assess the use of different anesthetic agents, including longer-acting local anesthetics and adjunct medications like dexamethasone or clonidine, to optimize analgesic duration and patient outcomes. Moreover, studies evaluating the functional recovery, rehabilitation progress, and long-term benefits of the NVL block compared to other techniques will provide valuable insights into its role in enhanced recovery protocols.

## Conclusions

The NVL block may serve as a potential alternative to the LFCN block for postoperative analgesia in knee surgeries, as both techniques provided comparable postoperative pain control and were safe and well tolerated, while the faster sensory onset and reduced opioid consumption associated with the NVL block did not translate into a meaningful improvement in pain control.

## Data Availability

Data is available on reasonable requests from the corresponding author.
